# The Smartphone Brain Scanner: A Portable Real-Time Neuroimaging System

**DOI:** 10.1371/journal.pone.0086733

**Published:** 2014-02-05

**Authors:** Arkadiusz Stopczynski, Carsten Stahlhut, Jakob Eg Larsen, Michael Kai Petersen, Lars Kai Hansen

**Affiliations:** Section for Cognitive Systems, DTU Compute, Technical University of Denmark, Kgs. Lyngby, Denmark; Universiteit Gent, Belgium

## Abstract

Combining low-cost wireless EEG sensors with smartphones offers novel opportunities for mobile brain imaging in an everyday context. Here we present the technical details and validation of a framework for building multi-platform, portable EEG applications with real-time 3D source reconstruction. The system – *Smartphone Brain Scanner* – combines an off-the-shelf neuroheadset or EEG cap with a smartphone or tablet, and as such represents the first fully portable system for real-time 3D EEG imaging. We discuss the benefits and challenges, including technical limitations as well as details of real-time reconstruction of 3D images of brain activity. We present examples of brain activity captured in a simple experiment involving imagined finger tapping, which shows that the acquired signal in a relevant brain region is similar to that obtained with standard EEG lab equipment. Although the quality of the signal in a mobile solution using an off-the-shelf consumer neuroheadset is lower than the signal obtained using high-density standard EEG equipment, we propose mobile application development may offset the disadvantages and provide completely new opportunities for neuroimaging in natural settings.

## Introduction

In the last few years, the research communities studying human behavior have gained access to unprecedented computational and sensing power that basically “fits into a pocket”. This has happened for both specialized equipment used for building research tools, such as Reality Mining Badges [1] or accelerometer sensors [2], and for consumer-grade, off-the-shelf devices. Smartphones and tablets are capable of sensing, processing, transmitting, and presenting information. This has already had a significant impact on many research domains, such as social science [3], computer-human interaction [4], and mobile sensing [5], [6]. In neuroscience there is a widely recognized need for mobility, i.e., for devices that support quantitative measurements in natural settings [7]–[9]. Here we present our work on the *Smartphone Brain Scanner*, investigate the feasibility of off-the-shelf, consumer-grade equipment in a neuroscience context, and build a mobile real-time platform for stimulus delivery, data acquisition, and processing with a focus on *real-time imaging* of brain activity.

Consumer-grade neuroheadsets, capable of recording brain activity generated by post-synaptic potentials of firing neurons, captured through electrodes placed on the scalp using Electroencepahlography (EEG), have only recently made mobile brain monitoring feasible. Seen from a mental state decoding perspective, even a single channel EEG recording measuring the changes in electrical potentials (based on a passive dry electrode positioned at the forehead and a reference typically placed on the earlobe), allows for measuring mental concentration and drowsiness by assessing the relative distribution of frequencies in brain-wave patterns throughout the day. Simply measuring the dynamic variability of brain-wave frequency components in a mobile scenario may be translated into neural signatures, e.g., reflecting whether a user is on the phone while driving a car [10]. Similarly, positioning the single EEG electrode headband over the temple may provide the foundation for building a Brain-Computer Interface (BCI) utilizing the ability to capture steady-state visual-evoked potentials (SSVEP) from the visual cortex when looking at flashing lights patterns, and thereby design a BCI interface for prediction with high accuracy and no previous training when a disabled user is focusing on a specific area of a screen, based on the time-locked EEG traces automatically generated as multiples of the particular flashing light frequencies [11].

As an example of the underlying technology used in several consumer products, the ThinkGear module manufactured by NeuroSky (http://www.neurosky.com/Products/ThinkGearAM.aspx) integrates a single dry electrode (reference and ground) attached to a headband. Essentially a system on a chip, it provides A/D conversion and amplification of one EEG channel, capable of capturing brain-wave patterns in the 3–100 Hz frequency range, recorded at 512 Hz sampling rate. Consumer neuroheadsets, such as those manufactured by Emotiv (http://www.emotiv.com) provide low-density neuroimaging based on 16 electrodes and typically support real-time signal processing in order to complement standard EEG measures with aggregate signals, which provide additional information on changes in mental state, or facilitate control of peripheral devices related to games. Their portability and built-in wireless transmission makes them suitable for the development of fully mobile systems, allowing for running EEG experiments in natural settings. The improved comfort of these mobile solutions also allows for extending neuroimaging experiments over several hours. Furthermore, the relatively low cost of the neuroheadsets and mobile devices potentially opens new opportunities for conducting novel types of social neuroscience experiments, where multiple subjects are monitored while they interact [12], [13].

However, such ‘low-fi’ mobile systems present a number of challenges. In real-time applications requiring signal processing to be performed with the lowest possible delay in order to present feedback to the user, the limited computational power of mobile devices may be a constraint. A solution might be to offload parts of the processing to an external server and retrieve the processed results over the network. This approach, however, requires network connection, possibly with low and constant delays, as well as more complicated client-server architecture. Also in terms of battery life, the local computation is more power-efficient than continuous transmission to the server and back. Consumer-grade mobile devices also present technical challenges for writing high-quality software; the devices operate on systems that are not real-time (RTOS), as they do not guarantee certain delays in data processing, and as such are ill-suited for time-sensitive tasks. These limitations might also affect timing of visual or auditory stimuli presentation, as well as synchronization with other sensors. From a neuroscience perspective, low-resolution recordings and artifacts induced in a mobile setup both present significant challenges. Noise and confounds are introduced by movement of the subject and electrical discharges, while the positioning of the electrodes might be less than ideal when compared to a standard laboratory EEG setup [14]–[17]. Nevertheless, we hold that these drawbacks are clearly offset by the advantages of being able to conduct studies incorporating larger groups of subjects over extended periods of time in more natural settings. We suggest that mobile EEG systems can be considered from two viewpoints: as stand-alone portable low-fi neuroimaging solutions, or as an add-on for retrieving neuroimaging data under natural conditions complementary to standard neuroimaging lab environments.

In terms of software programming, creating a framework for applications in C++ rather than in prevalent environments such as MATLAB, while approaching the problem as a smartphone sensing challenge, might enable new types of contributions to neuroscience. The Human-Computer Interaction (HCI) community is already starting to apply consumer-grade headsets to extend existing paradigms [18], thus incorporating neuroscience as a means to enhance data processing. Similarly, the availability of low-cost equipment means even general ‘hacker-and-tinkerers’ audiences will almost certainly gain interest in using neuroscience tools (http://neurogadget.com). We see a great value in the emerging potential of entirely new groups of researchers and developers becoming interested in neuroscience and obtaining tools allowing them to develop new kinds of applications.

In a previous communication [19], we discussed opportunities and challenges in mobile and portable EEG. Here we address the foundations of the Smartphone Brain Scanner system, focusing on engineering and technical aspects of both software and hardware components. We describe the computational architecture of the framework, and discuss timing, reliability, and quality of the obtained signal. In particular we report on the results of a validation experiment comparing the system with a conventional EEG acquisition system in a prototypical application.

### Related Work

Our real-time imaging EEG setup mediates between two hitherto disparate fields in sensorics, being on the one hand a down-sized neuroimaging device and on the other hand a sophisticated smartphone sensor system for cognitive monitoring in natural conditions. We therefore briefly review the state of the art in both domains.

#### Neuroimaging

Several software packages for offline and online analysis of biomedical and EEG signals are available. The most popular packages for off-line analysis are EEGLAB and FieldTrip; for building real-time BCI-oriented applications, notable frameworks are BCILAB, OpenViBE, and BCI2000.

EEGLAB is a toolbox for the MATLAB environment and is useful for processing collections of single-trial or averaged EEG data [20]. Functions available in this framework include data importing, preprocessing (artifact rejection, filtering), independent component analysis (ICA), and others. The framework can be used via a graphical interface or by directly manipulating MATLAB functions. The toolbox is available as an open source (GNU license) and can be extended to incorporate various EEG data formats coming from different hardware. Similarly, FieldTrip is an open source (GNU License) MATLAB toolbox for the analysis of MEG, EEG, and other electrophysiological data [21]. Among others, FieldTrip has pioneered high-quality source reconstruction methods for EEG imaging. FieldTrip has support for real-time processing of data based on a buffer construction that allows chunking of data for further processing in the MATLAB environment.

BCILAB is a toolbox for building online Brain-Computer Interface (BCI) models from available data [22]. It is a plugin for EEGLAB running in MATLAB, providing functionalities for designing, learning, use, and evaluation of real-time predictive models. BCILAB is focused on operating in real-time for detecting and classifying cognitive states. The classifier output from BCILAB can be streamed to a real-time application to effect stimulus or prosthetic control, or may be derived post-hoc from recorded data. The framework is extensible in various layers; additional EEG hardware as well as data processing steps (e.g., filters and classifiers) can be added. But as these toolboxes are developed within the MATLAB environment, neither FieldTrip's real-time buffer nor BCILAB are suitable for mobile application development.

OpenViBE is a software framework for designing, testing, and using Brain-Computer Interfaces [23]. The main application fields of OpenViBE are medical i.e., assistive technologies, bio- and neurofeedback as well as virtual reality multimedia applications. OpenViBE is an open source (LGPL 2.1) and targets an audience focused on building real-time applications for Windows and Linux Operating Systems, and does not specifically support light-weight mobile platforms. A similar C++ based framework for building real-time BCI applications is BCI2000 [24]. A comprehensive review of the BCI frameworks can be found in [25]. Some of the consumer EEG systems also include Software Development Kits (SDKs), allowing for data acquisition, processing, and building applications. Emotiv SDK, available with the Research Edition of the Emotiv system is multi-platform, currently running on Linux, OSX, and Windows. The SDK allows for building applications, either using raw EEG data or extracted features, including affective state and recognition of facial expressions based on eye movements. The extracted features can be integrated into a C++ or C# application through a set of dynamically linked libraries. Although such SDK frameworks can greatly speed up the process of building BCI applications, they are mostly targeted towards scenarios where immediate feedback is available, such as gaming, and it remains a challenge to validate or tweak code for custom needs. To sum up, none of the aforementioned software platforms can easily be adapted to support mobile and embedded devices.

There exist various repositories of openly available human EEG data [26], [27]. Such datasets contain both recordings from high-density and low-density systems and are an important tool for advancing the field. We feel that the increased availability of EEG systems will result in even more publicly available data. Although very beneficial for the field, this will undoubtedly raise concerns about the privacy of the subjects, whose very sensitive data in the form of EEG recordings, will possibly exist indefinitely.

#### Cognitive monitoring systems

Mobile brain imaging might also be viewed as yet another sensor extension to self-tracking applications, which have become prevalent with smartphones and the emergence of low-cost wearable devices - lowering the barriers for people to engage in life logging activities [28]. With the availability of multiple embedded sensors, modern smartphones have become a platform for out-of-the-box data acquisition of mobility (GPS, cellular network, WiFI), activity level (accelerometer), social interaction (Bluetooth, call, and text logs), and environmental context (microphone, camera, light sensor) [3]. Recently, non-invasive recording of brain activity has become common as several low-cost commercial EEG neuro-headset and headband systems have been made available, including the previously mentioned Emotiv EPOC and NeuroSky, the InteraXon Muse (http://www.interaxon.ca/), Axio (http://www.axioinc.com/), and Zeo (http://myzeo.com/). These sensors support applications ranging from BCI, game control, stress reduction, and cognitive training, to sleep monitoring. These neuroheadsets feature up to 16 electrodes, but ongoing developments promise next-generation low-cost EEG devices with a significantly higher number of electrodes, better quality signals, and improved comfort. The Smartphone Brain Scanner framework described in this paper can be used with mobile EEG devices with various numbers of electrodes to allow for capture of neuroimaging data over several hours. Battery tests on Samsung Galaxy Note with all wireless radios and screen turned off resulted in 11 hours of uninterrupted recording and storage of data from an Emotiv EPOC headset. However, current generation neuroheadsets are limited by their solution-based electrodes, which dry out. More comfortable designs [29], [30] may be required for continuous mobile neuroimaging throughout the day.

Beyond EEG, multiple bio signals and physiological parameters can contribute to cognitive state monitoring, such as respiratory rate [31], heart rate variability, galvanic skin response [32], blood pressure, oxygen saturation, body/skin temperature, ECG, EMG, and body movements [33]. A webcam or a camera embedded in a smartphone can allow measurements of heart rate, variability, and respiratory rate by analyzing the color channels in the video signal [34]. Continuous monitoring of heart rate is enabled by pulse watches (http://www.polar.com/) and recently by the Basis Band wrist-worn sensor (http://www.mybasis.com), which allows 24/7 recording under a subset of conditions (non-workout situations). Both continuous heart rate monitoring solutions allow user mobility and measurements in natural conditions. The Q Sensor from Affectiva (http://www.affectiva.com/) is an example of a system for monitoring galvanic skin response (GSR) and accelerometer and temperature data from a wrist-worn device. FitBit (http://www.fitbit.com/) is an example of a wearable pedometer, monitoring number of steps taken, distance traveled, calories burned, and floors climbed.

## Methods: Smartphone Brain Scanner

**Figure 1:**
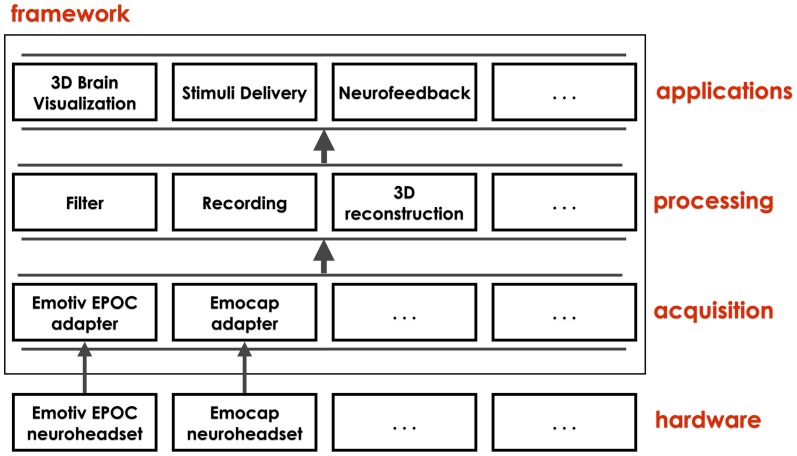
Smartphone Brain Scanner applications running on Android devices. Neurofeedback training and real-time 3D source reconstruction running on Android mobile devices via a wireless connection to an Emotiv or Easycap EEG systems.

The *Smartphone Brain Scanner* (SBS2) is a software platform for building research-oriented and end-user-oriented multi-platform EEG applications. The focus of the framework is on mobile devices (smartphones, tablets) and on consumer-grade (low-density and low-cost) mobile neurosystems (see Figure 1). The SBS2 is freely available under the MIT License on GitHub at https://github.com/SmartphoneBrainScanner. The repositories contain the core of the framework, as well as example applications. The documentation hosted on GitHub wiki pages (https://github.com/SmartphoneBrainScanner/smartphonebrainscanner2-core/wiki) includes instructions for compiling the software, building the hardware components, preparing the devices, and writing custom applications. An active mailing list for developers also exists at https://groups.google.com/forum/#forum/smartphonebrainscanner2-dev

**Figure 2:**
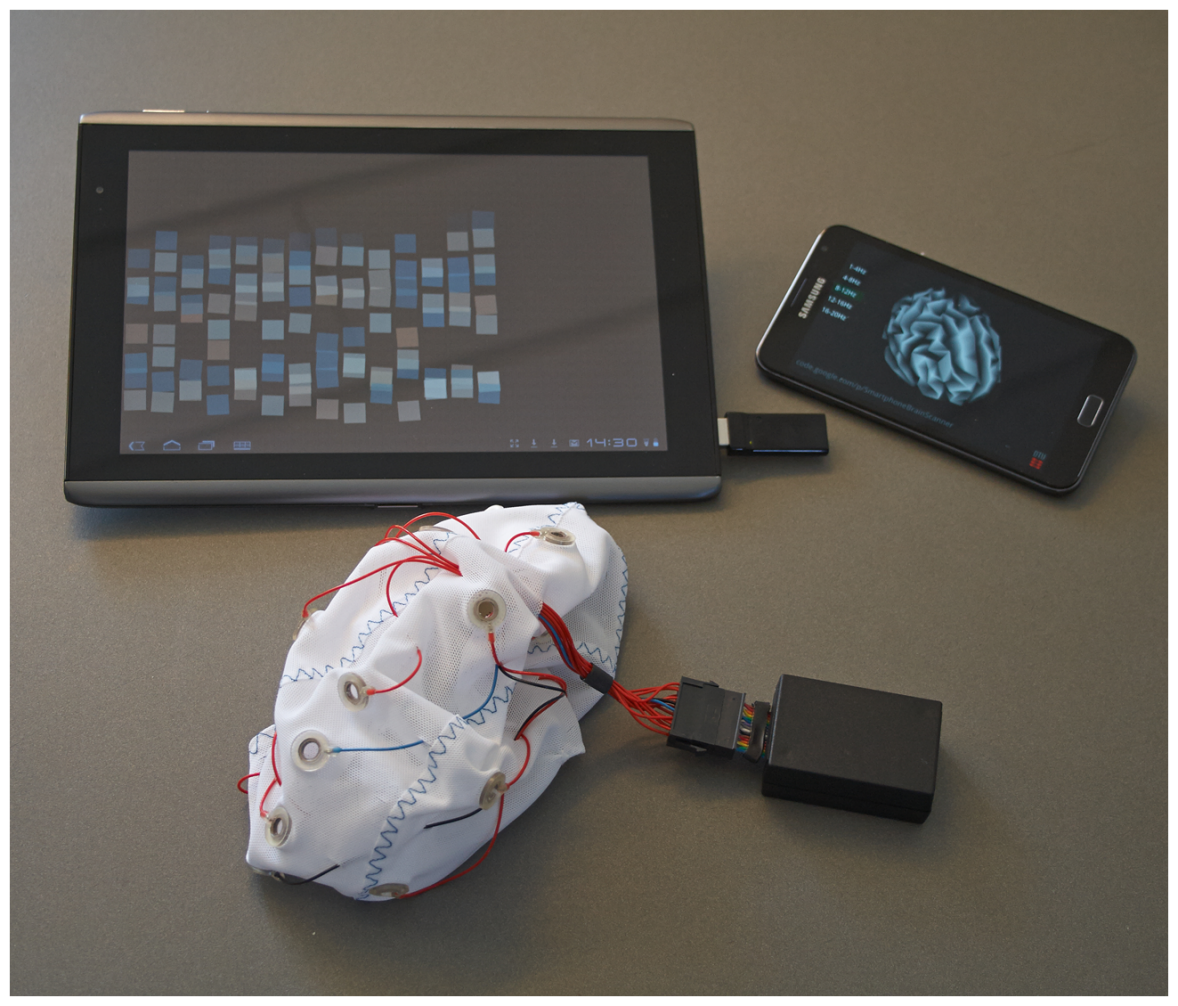
Overview of the layered architecture of the SBS2 framework. Data from the connected EEG hardware are acquired and extracted by specific adapters and all subsequent processing is hardware agnostic. The empty boxes indicate the extendability of the architecture allowing additional hardware devices for data acquisition and additional processing methods.

The SBS2 framework is divided into three layers: low-level data acquisition, data processing, and applications. The first two layers constitute the core of the system and include common elements used by various applications. An overview of the architecture is shown in Figure 2.

### Smartphone Brain Scanner

#### Key features

With a focus on the mobile devices, SBS2 is a multi-platform framework. The underlying technology – Qt – is an extension of C++ and is currently supported on the main desktop operating systems (Linux, OSX, Windows) as well as on mobile devices (Android, BB10, and partially iOS, see http://qt.digia.com/Product/Supported-Platforms/).

We have aimed for a modular framework, allowing for adding and modifying data acquisition and processing blocks. The modules are created as C++ classes and integrate directly with the core of the framework. The framework supports building real-time applications; data can be recorded for subsequent off-line analysis. However, most of the implemented data-processing blocks aim to provide real-time functionality for working with the EEG signal. The applications developed with SBS2 can be installed on both desktop and mobile devices; installation can be started by the user and distributed via regular channels, such as repositories and application stores.

#### Data acquisition

**Figure 3:**
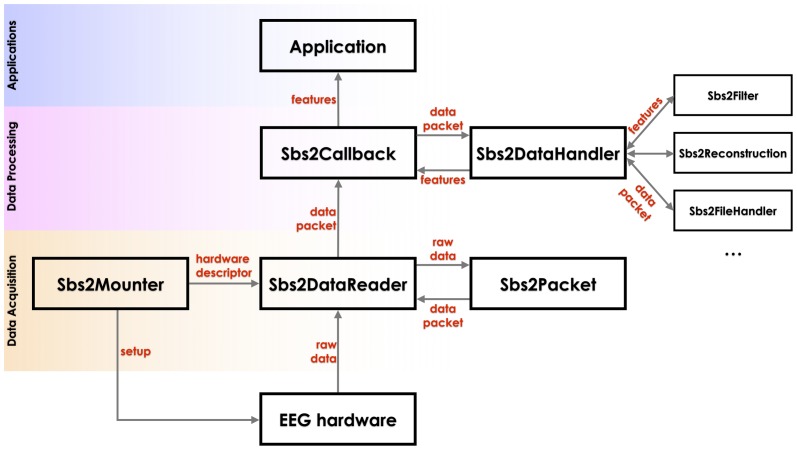
The Smartphone Brain Scanner architecture. Data are acquired in the first layer from the EEG hardware, passed to the Data Processing Layer, and extracted. Features, as well as raw values, are then available for applications.

The Data Acquisition layer is responsible for setting up communication with an EEG device, acquiring the raw data, and forming packets. Three primary objects are used: Sbs2Mounter, Sbs2DataReader, and Sbs2Packet, thereby abstracting all the specificities of the EEG systems (hardware) and of the OS+ device running the software (platform). Different embedded devices, even with the same OS, may require a specific code for certain low-level functionalities, for example to access the USB port. A higher-fidelity architecture is shown in Figure 3. The EEG hardware is set up by a specialized Sbs2Mounter object. The information about the hardware (e.g. mounting point, serial number) is passed to a Sbs2DataReader object. This object subsequently begins reading the raw data from the hardware. The raw data are passed to a Sbs2Packet object to create a proper encapsulation, setting the values for all the EEG channels and metadata. Once formed, the packet is pushed to the Data Processing layer via a Sbs2Callback object.

The Data Acquisition layer of the SBS2 was originally designed to support the Emotiv EEG headset. It has been extended to support additional hardware, such as custom made EasyCap hardware, by implementing additional classes of the hardware mounter, data reader, and packet creator. For Emotiv headset, this layer also contains the data decryption module, as the stream coming from the device is encrypted.

Mounting the EEG hardware on a desktop and embedded devices requires drivers, either standard kernel modules or proprietary drivers created by the vendor. The Emotiv EPOC USB receiver is mounted as*/dev/hidraw* in Linux (desktop and Android), provided the device and the kernel support the USB host mode and have the HIDRAW module enabled. Most desktop Linux flavors have both by default, but currently most Android mobile devices only support the USB host mode out-of-the-box. In the current implementation, a custom kernel needs to be compiled with the HIDRAW module enabled. Reading the data directly from the /dev/hidraw device requires ‘root’ privileges, which must be enabled on Android devices to acquire data from the Emotiv EPOC receiver. This is possible for most recent Android devices, e.g. for the Nexus (developer) line of devices. We can expect that the next generation of mobile neuroheadsets will use standardized Bluetooth low-energy protocols and Android devices will be able to support them by default. This will likely have a significant impact on the adoption of neuroimaging outside lab environments.

#### Data processing

Well-formed EEG packet objects are used for data processing. The functionality of this layer is hardware-agnostic and depends only on packet content, i.e. data for the EEG channels, reflecting a particular sensor configuration, and sampling frequency. Single packets are dispatched to different processing objects and methods, including recording, filtering, 3D reconstruction, etc. Some operations need to collect data into frames and run asynchronously (in separate thread), pushing the results back to the callback object once the results are ready.

Sbs2Callback is an object implementing the getData(Sbs2Packet*) method, to which single packets are always passed and can then be dispatched to the Sbs2DataHandler or pushed to the Application layer. Sbs2DataHandler is an object providing methods for data processing, by delegating them to specialized objects, including Sbs2FileHandler and Sbs2Filter.

The framework for data processing is extensible and new modules can be added to the core; the data handler prepares the data in a format expected by the processing block (e.g. collecting packets into larger frames) and runs the processing method. The currently implemented blocks allow for a variety of processing operations. The raw EEG data can be recorded, including time-stamped events (stimuli onsets, user responses, etc.). Raw packets, as well as extracted features and arbitrary values, can be streamed over the network for either data processing or interconnection between devices (multiplayer gaming is one example). Other methods for data processing, including filter, FFT, spatial filter (CSP), and classifier (LDA), are also implemented and can be used for building the pipelines.

### 3D Imaging

**Figure 4:**
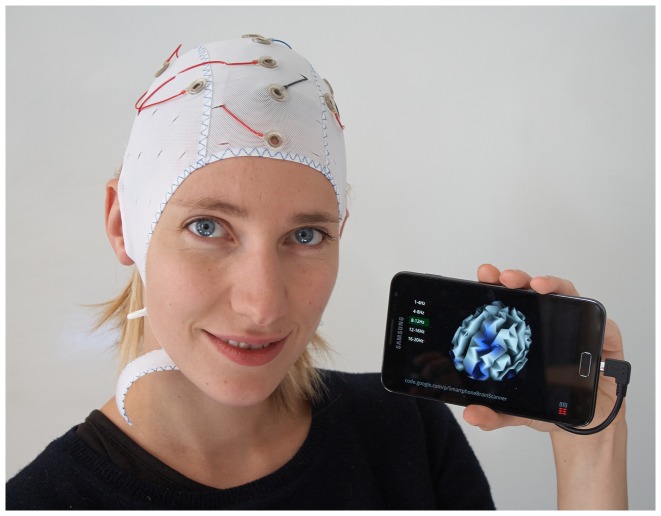
Snapshot of the SBS2 real time brain imaging system running on a Samsung Galaxy Note 2. EEG recorded using the Emocap [29], based on the Emotiv EEG wireless transmission setup. Visible in the picture is the entire setup required for data acquisition, processing, and visualization. The subject of the photograph has given written informed consent, as outlined in the PLOS consent form, to publish this photograph.

The most advanced data-processing block of the Smartphone Brain Scanner is the source reconstruction aimed at real-time 3D imaging as demonstrated in Figure 4. Videos demonstrating the Smartphone Brain Scanner are available at http://milab.imm.dtu.dk/eeg. Source reconstruction estimates the current sources within the brain most likely to have generated the observed EEG signal at scalp level. As the number of possible source locations far exceeds the number of channels, this is known to be an extremely ill-posed inverse problem. A unique solution is obtained by imposing prior information in correspondence with anatomical, physiological, or mathematical properties [35]–[37]. Implemented inverse methods in the SBS2 cover Bayesian formulations of the widely used Minimum-norm method (MN) [37] and low-resolution electromagnetic tomography (LORETA) [38]. The Bayesian formulation used in the SBS2 framework allows adaptation of hyper-parameters to different noise environments in real-time. This is an improvement over previous real-time source reconstruction approaches [39]–[41] that applied heuristics to estimate the parameters involved in the inverse method. The current source reconstruction is based on an assumed forward model matrix, 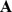, connecting scalp sensor signals 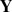 (channel by time) and current sources 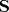 (cortical locations by time) [42]

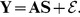
(1)The term 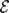 accounts for noise not modeled by the linear generative model. When estimating the forward model a number of issues are taken into consideration, such as sensor positions, the geometry of the head model (spherical or ‘realistic’ geometry), and tissue conductivity values [43]–[45]. With the forward model 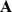 given and the linear relation in Eq. (1), the source generators can be estimated. We assume the noise term to be normally distributed, uncorrelated, and time-independent, which leads to the probabilistic formulation:
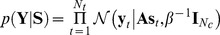
(2)

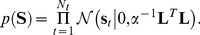
(3)Where 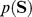 is the prior distribution over 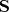 with 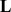 given as a graph Laplacian ensuring spatial coherence between sources and 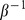 as the noise variance. Using Bayes' rule, the posterior distribution over the sources is maximized by
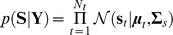


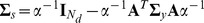


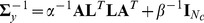
(4)

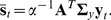
(5)Here, 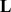 denotes a spatial coherence matrix, which in the current form takes advantage of the graph Laplacian using a fixed smoothness parameter (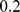).

Handling noise estimation is a crucial part for acquiring reliable source estimates. We have previously examined how eye-related artifacts can corrupt the source estimates for low density EEG caps with unevenly distributed sensors such as the Emotiv EPOC [19]. While we here have adopted the assumption of the noise to be uncorrelated, correlated noise can easily be included in the model above, either directly in the model or indirectly through pre-processing the data prior to the source modeling. Direct modeling of the correlated noise can be achieved by replacing the identity matrix 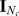 with a full noise covariance matrix 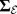. Estimation of the noise covariance matrix could e.g. be carried out through calibration sessions. By online estimating the hyperparameter 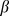 the inverse solver continuously can model the amount of noise present in the data.

The present data analytic pipeline does not include real-time artifact reduction steps, hence cleaning of data for eye, muscle, or motion induced artifacts must be carried out post hoc in the present system. Thus real-time imaging experiments, bio-feedback etc. should be done under circumstances that reduce artifacts.

## Methods: Experimental Designs

In this section we briefly describe the design of the experiments demonstrating and validating the potential of the SBS2 framework, the specific hardware, and the mobile approach in general.

### Timing and Data Quality

First, we analyzed the data and timing quality. Many neuroscience paradigms rely heavily on accurate synchronization between EEG signal and stimuli, user response, or data from other sensors (e.g., P300, steady state visual evoked potentials). However, we can also envision applications in which the present ‘low-cost’ mobile setup will be used to collect data from many subjects over extended periods, where precise synchronization is less important.

#### Emotiv EEG sampling

The measurements are all based on the Emotiv EEG neuroheadset. The nominal sampling frequency of this neuroheadset is 128 Hz (down-sampled from internal 2048 Hz). For validation purposes we tested the actual sampling rate obtained from three randomly picked Emotiv devices (10×10 min measurements for each).

#### Data quality

The Emotiv hardware adds a modulo 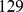 counter (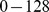) to every packet transmitted from the device. This allows for data quality control (dropped packets) with the accuracy of a modulo 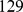. It is possible to obtain long recordings (over one hour) using this neuroheadset and SBS2. The battery in the Emotiv hardware is rated at 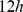 of continuous operation; in recording-only setup, a mobile device such as Galaxy Note (offline mode, screen off, only decrypting and recording) lasts for around 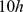. Provided good visibility between the Emotiv EEG neuroheadset transmitter (located in the back part of the headset) and the USB receiver was maintained, we were able to achieve zero packet loss in the full rundown recording. In order to acquire an EEG signal of good quality, the impedance between the electrodes and the scalp should be kept under 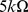. The Emotiv headset embeds the channel-quality information in the signal directly (2 Hz per channel, multiplexed into the signal). The values are unscaled, and come from applying a square wave of 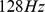 to the DRL feedback circuit and extracting the amplitude of the inherent square wave using phase-locked detection on each channel. In principle, the obtained values can be calibrated using a known impedance. For regular usage, however, the hardware manufacturer assures the green color of the indicator (channel quality value greater than 407) corresponds to sufficiently low impedance of the electrode. From our experience with the system this appears correct.

#### Timing

**Figure 5:**
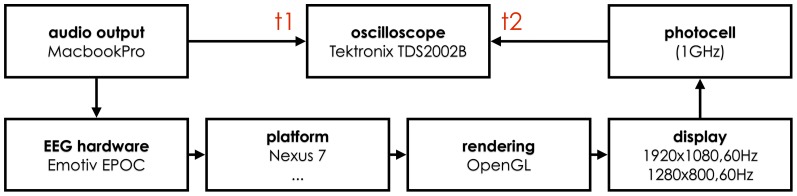
The timing measurement setup. 10 Hz sinusoid is generated with a computer sound card, amplified, and fed into an oscilloscope and the EEG hardware. The device acquiring the EEG data responds to the sinusoid signal with changes of screen brightness, which is detected by a photocell connected to the oscilloscope. The time difference between the two signals is used to calculate the system delay.

**Figure 6:**
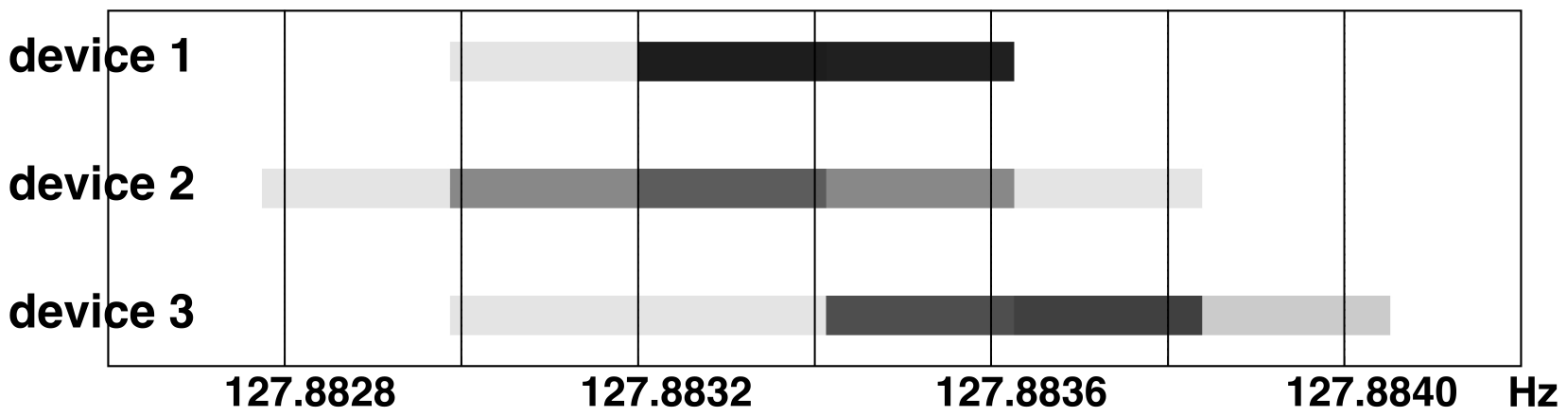
System response timings. The system responds to the sinusoid signal peak (time 0). The red color (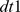) indicates minimal observed delay; the blue color (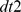) indicates jitter. Galaxy Note running Android 4.0.1, 60 Hz AMOLED screen, 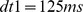, 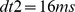; Nexus 7 running Android 4.1.1, 60 Hz IPS LCD screen, 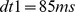, 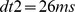; MacbookPro, LCD screen (60 Hz), 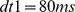, 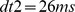.

In order to measure the total delay in the system, we used the setup as depicted in Figure 5. A sinusoidal audio tone of 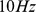, with its trailing and following periods of silence, was generated and amplified so it could be detected by the EEG hardware and also so it could be split into oscilloscope and EEG hardware. The software on the device performed peak detection on the signal and visualized the peaks by changing the screen color from black to white. This change was detected by a photocell, connected to the second channel of the oscilloscope. We can then calculate 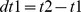, indicating the total delay of the system from the physical signal reaching the EEG hardware to being visualized on the screen (without any additional processing), see Figure 6. We also look at the jitter 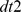 as the difference between 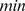 and 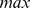 values of 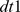. The observed delta depends on the EEG sampling rate (here 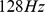), the processing power of the device, and the screen refresh rate (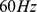 for all tested devices).

### Imagined Finger Tapping

One of the most widely investigated paradigms in the BCI literature is a task in which a subject is instructed to select between two or more different imagined movements [19], [46]–[49]. Such experiments are rooted in a central aim of many BCI systems, namely of being able to assist patients with severe motor disabilities to communicate by ‘thought’. In this contribution we replicated a classical experiment with imagined finger tapping (left vs. right) inspired by [49]. The setup consisted of a set of three different images with instructions: *Relax*, *Left*, *Right*. In order to minimize the effect of eye movements, the subject was instructed to focus on the center of the screen, where the instructions also appeared (3.5 inch display size, 800×480 pixels resolution, at a distance of 0.5 m). The instructions *Left* and *Right* appeared in random order. A total of 200 trials were conducted for a single subject.

## Results and Discussion

In this section we present and discuss the results of the experiments, validating the performance of the software, the platforms used, and the EEG hardware. These results aim to validate the underlying framework with respect to key engineering aspects and to outline the potential and limitations of the system, especially from the user and developer perspective. More complex experiments conducted using the system are described in [19].

### Timing and Data Quality

#### Emotiv EEG sampling

**Figure 7:**
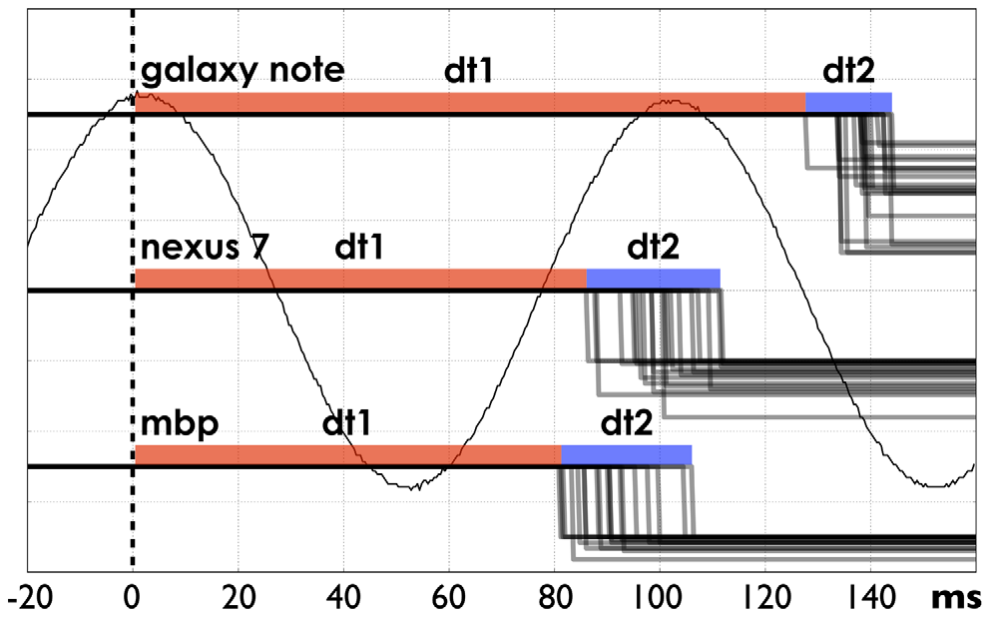
Measured sampling frequency, including measurement resolution for three random Emotiv EEG devices, 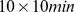 recordings for each. All measured rates, including uncertainty, are between 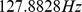 and 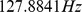, which corresponds to 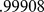 and 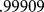 of nominal 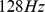. The measurements were performed with 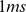 resolution (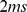 accuracy) on 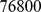 EEG packets. All tests were performed at normal temperature on a single day. We can note consistent results within and across devices.

From Figure 7 we can see that the Emotiv EPOC hardware a) has an actual sampling rate close to 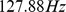 and b) keeps this sampling rate in a fairly consistent manner. Depending on the analysis performed on the data, one can assume 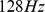, 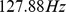, or measure the actual sampling rate for every Emotiv EPOC hardware device individually.

#### Timing

The results of the timing measurements (20 per device) are depicted in Figure 6.

We can see in the results for all devices that there is a significant delay between the signal reaching the EEG hardware and being fully processed in the software (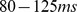). This delay, although significant, is fairly stable (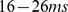 jitter) and thus can be corrected for.

**Figure 8:**
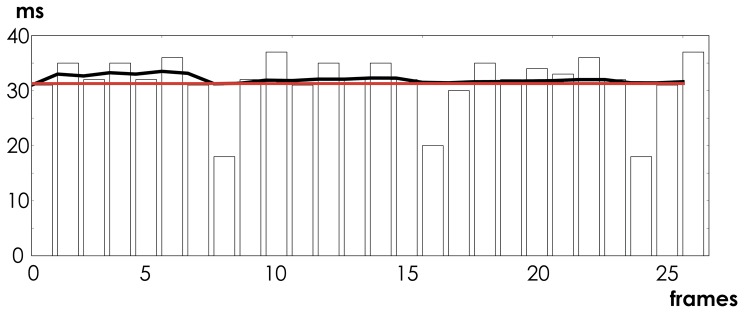
Distances between 4-sample frames. Red line indicates expected distance of 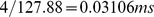 between the groups of four 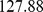 packets. The bars indicate the observed distance. We can see that the Emotiv system compensates every 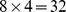 samples to keep the average (black line) at the correct level.

In the second set of measurements, we test the stability of the timing of the packets as they appear in the system. To measure this, we collect the packets from the Emotiv EPOC device and change the screen color every 4 packets (limited by screen refresh rate, 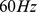). This change is then measured by a photocell, fed into the oscilloscope and the distance between the 4-packet packages is calculated. Figure 8 shows these measurements.

In summary, the stability and quality of the acquired signal is excellent. Most of the variations, including imperfect sampling rate or timing jitters, are constant and can largely be accounted for in the data analysis, if necessary.

#### 3D source reconstruction on-device performance

Source imaging was obtained using the Bayesian inverse solver for the linear model in Eq. (1). The forward matrix 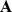 and cortical source mesh grid was based on a coarse resolution (5124 vertices) of the SPM8 template brain [50], further reduced to 1028 using Matlab's function reducepatch. We tested the performance of 3D reconstruction and hyper-parameters calculation on 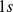 of raw EEG signal. The results on different platforms show the time needed for the actual reconstruction (fast) and update of hyper-parameters (slower): MacBookPro8,2 (Intel Core i7 Sandy Bridge 2.2 GHz): 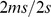, Nexus 7: 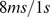, Galaxy Note: 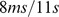, Acer Iconia: 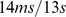. These results show that it is in fact possible to run 3D reconstruction of an EEG signal on mobile devices several times a second, and to update the hyper-parameters several times a minute.

### Imagined Finger Tapping – Online Source Reconstruction

In order to demonstrate the applicability of discriminating a simple task such as the left and right imagined finger tapping on the cortical source level in an online framework, the EEG data were acquired with the Emotiv EPOC neuroheadset and compared with EEG recordings acquired with a standard laboratory setting, viz. using 64 channels on a Biosemi Active-II device. The 64-channels were sub-sampled to represent the same channel locations as the Emotiv device.

**Figure 9:**
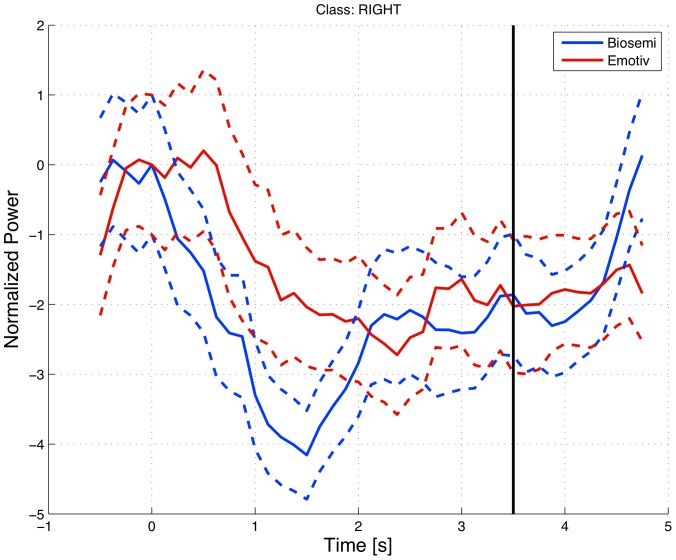
Finger-tapping results for Emotiv EEG and Biosemi standard equiment resampled to 14 channels. Mean (solid lines) and standard deviation (dashed lines) of reconstructed current source power in the left (L) Precentral AAL regions calculated across right-cued, imagined finger-tapping conditions. Mean activity was normalized to unit at 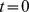. Both activities are based on 3D reconstruction with online estimation of the 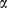 and 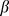 parameters using the Minimum Norm approch.

Imagined finger tapping is known to lead to a suppression of alpha (8–13 Hz) activity over the premotor/motor regions, with the contralateral areas normally being more desynchronized [51]. Thus, imagined right-finger tapping should lead to alpha activity being suppressed in the left pre-motor region. In Figure 9, we show the responses obtained with SBS2 and the standard equipment, demonstrating the framework's ability to reconstruct online meaningful current sources within the given region. In particular, Figure 9 shows how alpha power (8–13 Hz) is suppressed over time in the region of interest - Precentral Left AAL (Automated Anatomical Labeling). Both responses are calculated as the averaged response over 87 and 79 responses to ‘right imaging’ cued trials that remained after rejecting trials with artifacts. Note that, while the result is presented as an average over runs, the source localization was carried out in online mode with model parameters (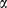 and 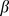) and current sources (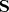) estimated online. We note the similarity of the suppression of the alpha power in the Left Precentral AAL region to imagined right-finger tapping trials as obtained by the Emotiv EPOC and the Biosemi system. The possible implications of using portable and low-cost systems such as *Smartphone Brain Scanner* in BCI context, together with more in-depth analysis of the finger tapping data are described in [19].

## Conclusions

We have presented the design, implementation, and evaluation of the first fully portable 3D EEG imaging system: The *Smartphone Brain Scanner*. The open source software allows real-time EEG data acquisition and source imaging on standard off-the-shelf Android mobile smartphones and tablets with a good spatial resolution and frame rates in excess of 40 fps. In particular, we have implemented a real-time solver for the ill-posed inverse problem with online Bayesian optimization of hyper-parameters (noise level and regularization).

The evaluation showed that the combined system provides for a stable imaging pipeline with a delay of 80–120 ms. We showed results of a cued, imagined finger-tapping experiment and compared the smartphone brain scanner's average power in the alpha band in a relevant motor area with that of conventional state-of-the-art laboratory equipment and found that these aggregate signals compare favorably with those obtained with standard equipment. Both show the expected de-synchronization on initiation of imagined motor actions.

The work presented here is extended in [19], where we discuss the perspectives and challenges of mobile and portable EEG systems. That work also includes results from more complex experiments, including neurofeedback applications and measuring emotional responses.

Future developments in hardware and software will allow for even better signal acquisition and analysis from low-density and mobile setups. This includes electrodes of different type and form (e.g. dry) and positioned in a non-standard way (e.g. inside ear canal). From the software perspective, more computation power available in the devices will allow for more powerful data processing and de-noising algorithms to be run (e.g. PCA-based or ICA-based artifacts rejection, more advanced 3D reconstruction), possibly using other available data sources (e.g. head movements obtained from gyroscopes). The present data analytic pipeline does not include real-time artifact reduction steps, hence cleaning of data for eye-, muscle-, or motion-induced artifacts must be carried out post hoc in the present system. Thus real-time imaging experiments, bio-feedback etc. should be done under circumstances that reduce artifacts.

We suggest the mobility and simplified application development may enable completely new research directions for imaging neuroscience and thus offset the expected reduced signal quality of a mobile off-the-shelf, low-density neuroheadset relative to more conventional and controlled, high-density laboratory equipment.

## References

[1] Choudhury T, Pentland A (2003) Sensing and modeling human networks using the sociometer. In: Proc. the 7th IEEE International Symposium on Wearable Computers (ISWC2003). pp. 216–222.

[2] Van Laerhoven K, Cakmakci O (2000) What shall we teach our pants? In: Wearable Computers, The Fourth International Symposium on. IEEE, pp. 77–83.

[3] Aharony N, Pan W, Ip C, Khayal I, Pentland A (2011) Social fmri: Investigating and shaping social mechanisms in the real world. Pervasive and Mobile Computing 7: 643–659.

[4] Brown B, Reeves S, Sherwood S (2011) Into the wild: challenges and opportunities for field trial methods. In: Proceedings of the SIGCHI Conference on Human Factors in Computing Systems. ACM, pp. 1657–1666.

[5] Jensen B, Larsen JE, Jensen K, Larsen J, Hansen LK (2010) Estimating human predictability from mobile sensor data. In: Machine Learning for Signal Processing (MLSP), 2010 IEEE International Workshop on. IEEE, pp. 196–201.

[6] Kwok R (2009) Personal technology: Phoning in data. Nature 458: 959. 19396118 10.1038/458959a

[7] Makeig S, Gramann K, Jung T, Sejnowski T, Poizner H (2009) Linking brain, mind and behavior. International Journal of Psychophysiology 73: 95–100. 19414039 10.1016/j.ijpsycho.2008.11.008 PMC2796545

[8] Blankertz B, Tangermann M, Vidaurre C, Fazli S, Sannelli C, et al (2010) The berlin brain-computer interface: non-medical uses of bci technology. Frontiers in Neuroscience 4. 10.3389/fnins.2010.00198 PMC3002462 21165175

[9] Gramann K, Gwin J, Ferris D, Oie K, Jung T, et al (2011) Cognition in action: imaging brain/body dynamics in mobile humans. Reviews in the Neurosciences 22: 593–608. 22070621 10.1515/RNS.2011.047

[10] Yasui Y (2009) A brainwave signal measurement and data processing technique for daily life applications. Journal of Physiological Anthropology 28: 145–150. 19483376 10.2114/jpa2.28.145

[11] Luo A, Sullivan T (2010) A user-friendly ssvep-based brain-computer interface using a time-domain classifier. Journal of Neural Engineering 7: 026010. 10.1088/1741-2560/7/2/026010 20332551

[12] Konvalinka I, Roepstorff A (2012) The two-brain approach: how can mutually interacting brains teach us something about social interaction? Frontiers in Human Neuroscience 6. 10.3389/fnhum.2012.00215 PMC3402900 22837744

[13] Dumas G (2011) Towards a two-body neuroscience. Communicative & Integrative Biology 4: 349–352. 21980578 10.4161/cib.4.3.15110 PMC3187906

[14] Stahlhut C, Attias H, Stopczynski A, Petersen M, Larsen JE, et al.. (2012) An evaluation of EEG scanner's dependence on the imaging technique, forward model computation method, and array dimensionality. In: 34th Annual International Conference of the IEEE Engineering in Medicine and Biology Society. pp. 1–4. 10.1109/EMBC.2012.6346235 23366196

[15] Chi Y, Wang YT, Wang Y, Maier C, Jung TP, et al (2012) Dry and noncontact eeg sensors for mobile brain-computer interfaces. IEEE Transactions on Neural Systems and Rehabilitation Engineering 20: 228–235. 22180514 10.1109/TNSRE.2011.2174652

[16] Gramann K, Gwin JT, Bigdely-Shamlo N, Ferris DP, Makeig S (2010) Visual evoked responses during standing and walking. Frontiers in Human Neuroscience 10.3389/fnhum.2010.00202 PMC3024562 21267424

[17] Gwin JT, Gramann K, Makeig S, Ferris DP (2010) Removal of movement artifact from high-density eeg recorded during walking and running. Journal of Neurophysiology 10.1152/jn.00105.2010 PMC3774587 20410364

[18] Vi C, Subramanian S (2012) Detecting error-related negativity for interaction design. In: Proceedings of the 2012 ACM annual conference on Human Factors in Computing Systems. ACM, pp. 493–502.

[19] Stopczynski A, Stahlhut C, Petersen MK, Larsen JE, Jensen CF, et al (2013) Smartphones as pocketable labs: Visions for mobile brain imaging and neurofeedback. International Journal of Psychophysiology Available: 10.1016/j.ijpsycho.2013.08.007. 23994206

[20] Delorme A, Makeig S (2004) Eeglab: an open source toolbox for analysis of single-trial eeg dynamics including independent component analysis. Journal of Neuroscience Methods 134: 9–21. 15102499 10.1016/j.jneumeth.2003.10.009

[21] Oostenveld R, Fries P, Maris E, Schoffelen J (2011) Fieldtrip: open source software for advanced analysis of meg, eeg, and invasive electrophysiological data. Computational Intelligence and Neuroscience 2011: 1. 21253357 10.1155/2011/156869 PMC3021840

[22] Delorme A, Mullen T, Kothe C, Acar Z, Bigdely-Shamlo N, et al (2011) Eeglab, sift, nft, bcilab, and erica: new tools for advanced eeg processing. Computational Intelligence and Neuroscience 2011: 10. 10.1155/2011/130714 PMC3114412 21687590

[23] Renard Y, Lotte F, Gibert G, Congedo M, Maby E, et al (2010) Openvibe: an open-source software platform to design, test, and use brain-computer interfaces in real and virtual environments. Presence: Teleoperators and Virtual Environments 19: 35–53.

[24] Schalk G, McFarland D, Hinterberger T, Birbaumer N, Wolpaw J (2004) Bci2000: a generalpurpose brain-computer interface (bci) system. IEEE Transactions on Biomedical Engineering 51: 1034–1043. 15188875 10.1109/TBME.2004.827072

[25] Brunner C, Andreoni G, Bianchi L, Blankertz B, Breitwieser C, et al (2011) Bci software platforms. Towards Practical Brain-Computer Interfaces 303–331.

[26] Dtu compute neuro wiki. Available: http://neuro.compute.dtu.dk/wiki/Electroencephalography#Data. Accessed 2013 Feb 27.

[27] Ucsd publicly available eeg data. Available: http://sccn.ucsd.edu/ãrno/fam2data/publicly available EEG data.html. Accessed 2013 Feb 27.

[28] Swan M (2012) Sensor mania! the internet of things, wearable computing, objective metrics, and the quantified self 2.0. Journal of Sensor and Actuator Networks 1: 217–253.

[29] Debener S, Minow F, Emkes R, Gandras K, Vos M (2012) How about taking a low-cost, small, and wireless eeg for a walk? International Journal of Psychophysiology 49: 1617–1621. 10.1111/j.1469-8986.2012.01471.x 23013047

[30] Looney D, Kidmose P, Park C, Ungstrup M, Rank ML, et al (2012) The in-the-ear recording concept: User-centered and wearable brain monitoring. Pulse, IEEE 3: 32–42. 10.1109/MPUL.2012.2216717 23247157

[31] Moraveji N, Adiseshan A, Hagiwara T (2012) Breathtray: augmenting respiration self-regulation without cognitive deficit. In: CHI '12 Extended Abstracts on Human Factors in Computing Systems. New York, NY, USA: ACM, CHI EA '12, pp. 2405–2410. doi:10.1145/2212776.2223810. Available: http://doi.acm.org/10.1145/2212776.2223810.

[32] Poh M, Swenson N, Picard R (2010) A wearable sensor for unobtrusive, long-term assessment of electrodermal activity. IEEE Transactions on Biomedical Engineering 57: 1243–1252. 20172811 10.1109/TBME.2009.2038487

[33] Pantelopoulos A, Bourbakis N (2010) A survey on wearable sensor-based systems for health monitoring and prognosis. IEEE Transactions on Systems, Man, and Cybernetics, Part C: Applications and Reviews 40: 1–12.

[34] Poh M, McDuff D, Picard R (2011) Advancements in noncontact, multiparameter physiological measurements using a webcam. IEEE Transactions on Biomedical Engineering 58: 7–11. 20952328 10.1109/TBME.2010.2086456

[35] Baillet S, Garnero L (1997) A bayesian approach to introducing anatomo-functional priors in the EEG/MEG inverse problem. IEEE Transactions on Biomedical Engineering 44: 374–385. 9125822 10.1109/10.568913

[36] Phillips C, Rugg M, Friston K (2002) Anatomically Informed Basis Functions for EEG Source Localisation: Combining Functional and Anatomical Constraints. NeuroImage 16: 678–695. 12169252 10.1006/nimg.2002.1143

[37] Hämäläinen M, Ilmoniemi R (1994) Interpreting magnetic fields of the brain: minimum norm estimates. Medical & Biological Engineering & Computing 32: 35–42. 8182960 10.1007/BF02512476

[38] Pascual-Marqui RD, Michel CM, Lehmann D (1994) Low resolution electromagnetic tomography: a new method for localizing electrical activity in the brain. International Journal of Psychophysiology 18: 49–65. 7876038 10.1016/0167-8760(84)90014-x

[39] Congedo M, Lotte F, Lécuyer A (2006) Classification of movement intention by spatially filtered electromagnetic inverse solutions. Physics in Medicine and Biology 51: 1971–1989. 16585840 10.1088/0031-9155/51/8/002

[40] Noirhomme Q, Kitney R, Macq B (2008) Single-trial eeg source reconstruction for brain-computer interface. IEEE Transactions on Biomedical Engineering 55: 1592–1601. 18440905 10.1109/TBME.2007.913986

[41] Besserve M, Martinerie J, Garnero L (2011) Improving quantification of functional networks with eeg inverse problem: Evidence from a decoding point of view. NeuroImage 55: 1536–1547. 21276859 10.1016/j.neuroimage.2011.01.056

[42] Baillet S, Mosher J, Leahy R (2001) Electromagnetic brain mapping. IEEE Signal Processing Magazine 18: 14–30.

[43] Wolters C, Kostler H, Moller C, Hardtlein J, Grasedyck L, et al (2007) Numerical mathematics of the subtraction method for the modeling of a current dipole in EEG source reconstruction using finite element head models. SIAM J Sci Comp 30: 24–45.

[44] Hallez H, Vanrumste B, Grech R, Muscat J, De Clercq W, et al (2007) Review on solving the forward problem in EEG source analysis. Journal of Neuroengineering and Rehabilitation 4: 46. 18053144 10.1186/1743-0003-4-46 PMC2234413

[45] Drechsler F, Wolters C, Dierkes T, Si H, Grasedyck L (2009) A full subtraction approach for finite element method based source analysis using constrained Delaunay tetrahedralisation. NeuroImage 46: 1055–1065. 19264145 10.1016/j.neuroimage.2009.02.024

[46] Müller-Gerking J, Pfurtscheller G, Flyvbjerg H (1999) Designing optimal spatial filters for singletrial EEG classification in a movement task. Clinical Neurophysiology 110: 787–798. 10400191 10.1016/s1388-2457(98)00038-8

[47] Babiloni F, Cincotti F, Lazzarini L, Millan J, Mourino J, et al (2000) Linear classification of low-resolution eeg patterns produced by imagined hand movements. IEEE Transactions on Rehabilitation Engineering 8: 186–188. 10896181 10.1109/86.847810

[48] Dornhege G, Blankertz B, Curio G, Muller K (2004) Boosting bit rates in non-invasive EEG single-trial classifications by feature combination and multi-class paradigms. IEEE Transactions on Biomedical Engineering 51 6: 993–1002. 15188870 10.1109/TBME.2004.827088

[49] Blankertz B, Dornhege G, Krauledat M, Muller K, Kunzmann V, et al (2006) The berlin braincomputer interface: Eeg-based communication without subject training. IEEE Transactions on Neural Systems and Rehabilitation Engineering 14: 147–152. 16792281 10.1109/TNSRE.2006.875557

[50] Litvak V, Mattout J, Kiebel S, Phillips C, Henson R, et al (2011) Eeg and meg data analysis in spm8. Computational Intelligence and Neuroscience 2011 10.1155/2011/852961 PMC3061292 21437221

[51] Pfurtscheller G, Lopes da Silva F (1999) Event-related eeg/meg synchronization and desynchronization: basic principles. Clinical Neurophysiology 110: 1842–1857. 10576479 10.1016/s1388-2457(99)00141-8

