# Correction: The Smartphone Brain Scanner: A Portable Real-Time Neuroimaging System

**DOI:** 10.1371/journal.pone.0096652

**Published:** 2014-04-25

**Authors:** The PLOS ONE Staff

The images for Figures 1 and 2 are incorrectly switched with each other, and the images for Figures 6 and 7 are incorrectly switched with each other. The figure legends appear in the correct order. Please see the correct figures here.

**Figure 1:**
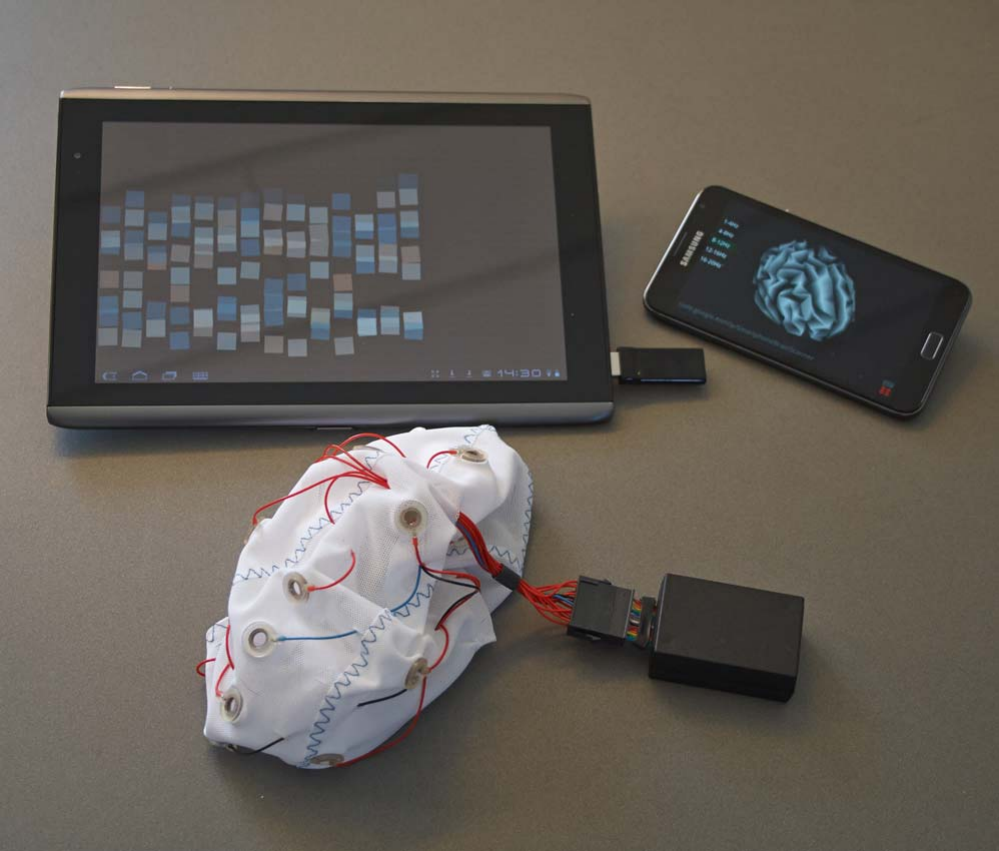
Smartphone Brain Scanner applications running on Android devices. Neurofeedback training and real-time 3D source reconstruction running on Android mobile devices via a wireless connection to an Emotiv or Easycap EEG systems.

**Figure 2:**
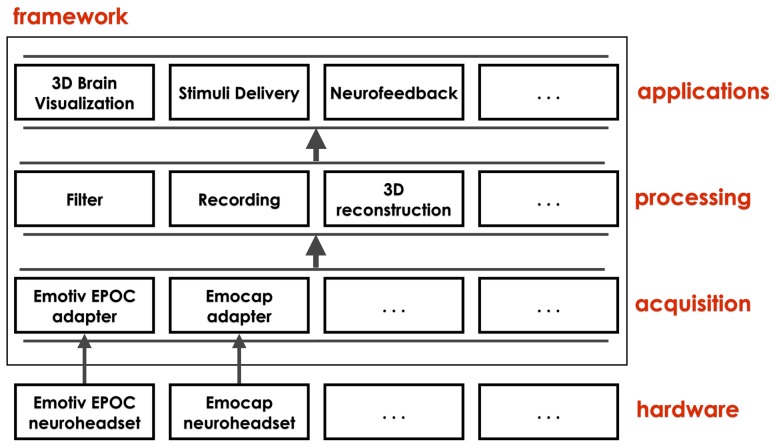
Overview of the layered architecture of the SBS2 framework. Data from the connected EEG hardware are acquired and extracted by specific adapters and all subsequent processing is hardware agnostic. The empty boxes indicate the extendability of the architecture allowing additional hardware devices for data acquisition and additional processing methods.

**Figure 6:**
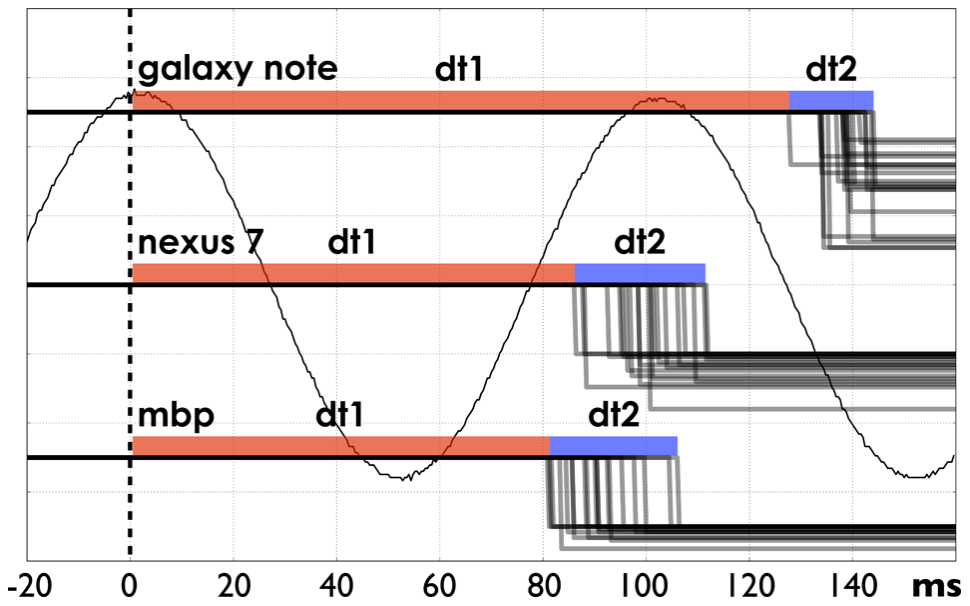
System response timings. The system responds to the sinusoid signal peak (time 0). The red color (*dt*1) indicates minimal observed delay; the blue color (*dt*2) indicates jitter. Galaxy Note running Android 4.0.1, 60 Hz AMOLED screen, *dt*1  =  125*ms*, *dt*2  =  16*ms*; Nexus 7 running Android 4.1.1, 60 Hz IPS LCD screen, *dt*1  =  85*ms*, *dt*2  =  26*ms*; MacbookPro, LCD screen (60 Hz), *dt*1  =  80*ms*, *dt*2  =  26*ms*.

**Figure 7:**
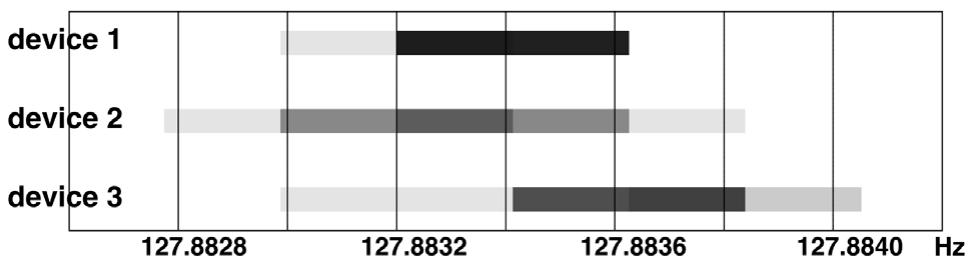
Measured sampling frequency, including measurement resolution for three random Emotiv EEG devices, 10 × 10min recordings for each. All measured rates, including uncertainty, are between 127.8828*Hz* and 127.8841*Hz*, which corresponds to .99908 and .99909 of nominal 128*Hz*. The measurements were performed with 1*ms* resolution (2*ms* accuracy) on 76800 EEG packets. All tests were performed at normal temperature on a single day. We can note consistent results within and across devices.

## References

[1] Stopczynski A, Stahlhut C, Larsen JE, Petersen MK, Hansen LK (2014) The Smartphone Brain Scanner: A Portable Real-Time Neuroimaging System. PLoS ONE 9(2): e86733 doi:10.1371/journal.pone.0086733 24505263 10.1371/journal.pone.0086733 PMC3914802

